# Regulating Axonal Responses to Injury: The Intersection between Signaling Pathways Involved in Axon Myelination and The Inhibition of Axon Regeneration

**DOI:** 10.3389/fnmol.2016.00033

**Published:** 2016-06-08

**Authors:** Sudheendra N. R. Rao, Damien D. Pearse

**Affiliations:** ^1^The Miami Project to Cure Paralysis, University of Miami Miller School of MedicineMiami, FL, USA; ^2^The Department of Neurological Surgery, University of Miami Miller School of MedicineMiami, FL, USA; ^3^The Neuroscience Program, University of Miami Miller School of MedicineMiami, FL, USA; ^4^The Interdisciplinary Stem Cell Institute, University of Miami Miller School of MedicineMiami, FL, USA; ^5^Bruce W. Carter Department of Veterans Affairs Medical CenterMiami, FL, USA

**Keywords:** myelination, axon regeneration, spinal cord injuries, Schwann cell, oligodendrocytes, signaling pathways, radial growth, adaptive myelination

## Abstract

Following spinal cord injury (SCI), a multitude of intrinsic and extrinsic factors adversely affect the gene programs that govern the expression of regeneration-associated genes (RAGs) and the production of a diversity of extracellular matrix molecules (ECM). Insufficient RAG expression in the injured neuron and the presence of inhibitory ECM at the lesion, leads to structural alterations in the axon that perturb the growth machinery, or form an extraneous barrier to axonal regeneration, respectively. Here, the role of myelin, both intact and debris, in antagonizing axon regeneration has been the focus of numerous investigations. These studies have employed antagonizing antibodies and knockout animals to examine how the growth cone of the re-growing axon responds to the presence of myelin and myelin-associated inhibitors (MAIs) within the lesion environment and caudal spinal cord. However, less attention has been placed on how the myelination of the axon after SCI, whether by endogenous glia or exogenously implanted glia, may alter axon regeneration. Here, we examine the intersection between intracellular signaling pathways in neurons and glia that are involved in axon myelination and axon growth, to provide greater insight into how interrogating this complex network of molecular interactions may lead to new therapeutics targeting SCI.

## Myelinating Glia of the CNS and PNS

Neuron-glia interactions have been fundamental to the structure and function of the brain throughout evolution (Herculano-Houzel, 2014). Oligodendrocytes (OLs) in the central nervous system (CNS) and Schwann cells (SCs) in the peripheral nervous system (PNS) ensheathe axons with myelin for the promotion of saltatory conduction (Nave and Werner, 2014). In the case of OLs, they extend their processes spirally inward, around the axons, in a corkscrew-like manner to lay down a multi-lamellar, compact, lipid rich sheath (myelin sheath; myelin from myelós, Greek for marrow) on the axons. Formation of the myelin sheath occurs in an outside to inside fashion by a process involving homotypic fusion of myelinophore organelles within the confines of their processes (Ioannidou et al., 2012; Snaidero et al., 2014; Szuchet et al., 2015). OLs represent almost 75% of the neocortical glial population, and each OL is capable of laying down myelin on 40–60 short axonal segments of multiple CNS axons with varying diameter (Matthews and Duncan, 1971; Lubetzki et al., 1993; Shaham, 2006; Pelvig et al., 2008; Fields et al., 2015). In the CNS, the renewal of myelinating OLs comes from oligodendrocyte precursor cells (OPCs). OPCs, activated by specific mitogens [e.g., platelet derived growth factor (PDGF) and neurotrophin-3 (NT3)], and differentiating factors [e.g., thyroid hormone T3, insulin growth factor-1 (IGF-1), transforming growth factor-β1 (TGF-β1) and stromal derived factor-1 (SDF-1)] proliferate and progress through a pre-myelinating phase to eventually become myelinating OLs (Boulanger and Messier, 2014). CNS myelination begins prenatally and proceeds gradually at the level of axonal tracts in a rostral to caudal—dorsal to ventral gradient (Schreyer and Jones, 1982; Almeida et al., 2011; Wang and Young, 2014). In mice, the generation of a surprisingly large numbers of OLs occurs even in adulthood, which contribute towards a distinct remodeling of the myelin sheath and remyelination after CNS injury (Powers et al., 2013; Young et al., 2013). Studies have also suggested that, in humans, neocortical myelination is protracted, lending support to the idea that the presence of myelination and remyelination in the adult is relevant across species (Miller et al., 2012; Glasser et al., 2014; Shafee et al., 2015). Accumulating evidence suggests that not only do actively growing axons become myelinated, but also axons which have reached their target can undergo continual myelin remodeling, a capability that persists when the axon is injured (Yeung et al., 2014).

In the PNS, SCs are derived from neural crest cells that pass through precursor and immature stages to eventually become myelinating or non-myelinating SCs (Jessen and Mirsky, 2005). In rodents, Schwann cell precursors (SCPs) are observed in spinal nerves by E12-E14 (Jessen et al., 1994; Dong et al., 1999). Survival of SCPs usually requires growth factors like PDGF, NT-3, endothelin, fibroblast growth factor (FGF) and IGF (Woodhoo et al., 2004). SCPs continue to proliferate under mitogens such as axonal neuregulin 1 (NRG1) and TGFβ to become immature SCs that envelop a large group of axons *en masse* (Ridley et al., 1989; Morrissey et al., 1995; Woodhoo and Sommer, 2008). Axonal caliber and glia-axonal contact are critical in deciding the myelinating and non-myelinating, inter-convertible fates of SCs (Weinberg and Spencer, 1975; Aguayo et al., 1976; Trapp et al., 1988; Voyvodic, 1989; LeBlanc and Poduslo, 1990). Through the process of radial sorting, that continues postnatally, immature SCs differentiate and establish a 1:1 relationship with peripheral axons and spirally ensheathe and myelinate large diameter axons, whereas some mature SCs, termed Remak cells, remain associated with multiple, small diameter axons without myelinating them (Feltri et al., 2015).

Myelination is a multistage process with considerable overlap among its different phases. In general, these phases involve: (1) the migration and ensuing differentiation of glial precursors into mature myelinating glia; (2) the initial recognition of the axon, axon-glia contact, axonal segment selection and subsequent ensheathment of the target axonal segments by the myelinating glia; (3) the initiation of myelin-associated protein expression in the myelinating glia and finally; (4) the compaction and maturation of the myelin sheath (Szuchet et al., 2015). Further fine-tuning of the myelination process involves the generation of functional axonal domains such as nodes of Ranvier, paranodes and juxtaparanodes.

There is a striking difference, however, in the structural proteins that make up the myelin of the CNS and the PNS. CNS myelin produced by OLs is compact, rich in glycolipid (e.g., galactocerebroside) and sulfolipid-sulfatide, has a higher concentration of proteolipid protein (PLP) and consists of unique glycoproteins, such as the myelin-associated inhibitors (MAIs) including myelin oligodendrocyte glycoprotein (OMgP/MOG; Nave and Trapp, 2008; Jahn et al., 2009). In contrast, myelin protein zero (P0/MPZ) and peripheral myelin protein (PMP22) constitute characteristic structural proteins of peripheral myelin (Patzig et al., 2011). Despite these structural and composition differences, axonal signaling plays an important role in the regulation of both OL and SC development, myelin biogenesis and their ability to myelinate CNS and the PNS axons, respectively (Barres and Raff, 1999; Nave and Trapp, 2008; Taveggia et al., 2010). In humans, OPC maturation takes place almost 3 months before the onset of myelination (around 40 weeks), reiterating the need for specialized signaling mechanisms between OLs and axons for the initiation of myelination (Brody et al., 1987; Kinney et al., 1988; Back et al., 2002). In contrast, SCPs and immature SCs appear at around 12 weeks of fetal development, and mature SCs commence peripheral myelination 2 weeks later, first at the motor roots, then the sensory roots (Cravioto, 1965). Most of the peripheral myelination completes within 1 year of birth, whereas CNS myelination continues well past the first decade of life (Jakovcevski et al., 2009; Bercury and Macklin, 2015).

Injury to CNS axons, in contrast to that of PNS axons, leads to impaired axonal regeneration as a result of the actions of various intrinsic and extrinsic factors (Afshari et al., 2009). These factors adversely affect the gene programs that govern the expression of regeneration-associated genes (RAGs) and the production of a diversity of extracellular matrix molecules (ECMs), leading to structural alterations in the axon that perturb the axonal growth machinery or lead to the formation of extraneous barriers to axonal regeneration at the site of lesion (Kaplan et al., 2015). Here, the role of myelin (both intact and debris) in altering injured axon growth responses has been the focus of both targeted therapeutic approaches and transgenic mouse studies, in which components of myelin, specifically MAIs, have been blocked, or are genetically knocked out (Raisman, 2004; Schwab and Tuszynski, 2010; Lee and Zheng, 2012). However, there has been less attention on how myelination of the injured axon, whether by endogenous or exogenously transplanted glia as a therapeutic approach, may alter axon regeneration. Combinatorial approaches involving the modulation of the: (1) properties of glial scar; and (2) MAI signaling and transplantation of myelination-competent cells, with or without trophic factors, have all yielded limited axonal regeneration caudal to the injury site in various spinal cord injury (SCI) models (Deumens et al., 2005). Understanding the pathways involved in myelination and how these pathways may directly play a role in or intersect with, signaling cascades involved in axon growth or its inhibition, may provide new avenues for developing regenerative therapies after CNS injury. The current review examines the distinct signaling pathways implicated in axon-glia communication during myelination, and discusses how these same pathways play a role in altering axonal growth responses after injury.

## The Intersection of Signaling Pathways Regulating Myelination and Axonal Growth

### Notch Signaling

Notch (notch1 and notch2) is a transmembrane receptor and Delta, Delta-like (Dll-1, 3, 4), Serrate/Jagged (jagged 1, jagged 2), F3/Contactin and NB3 (Contactin-6) are its known ligands (Andersson et al., 2011). Notch ligands that are present on axons play an instructive role in the development of various glia, including OLs (Gaiano and Fishell, 2002; Givogri et al., 2002; Stump et al., 2002), and regulate the differentiation of SCPs and the proliferation of SCs, but postnatally can also act in an inhibitory fashion towards SC mediated myelination (Woodhoo et al., 2009). Axons harbor extracellular notch ligands jagged1, F3/contactin and NB3 near the paranodes, whereas OLs and SCs express the notch receptors 1 and 2 (Stidworthy et al., 2004; Woodhoo et al., 2009). Adult rodent and human brain shows expression of notch receptors (notch1, notch2) and its ligand (jagged 1; Berezovska et al., 1998; Stump et al., 2002; Chen et al., 2005). Axonal jagged1 blocks OPC differentiation into OLs within the adult rodent brain (Grandbarbe et al., 2003; Park and Appel, 2003). However, evidence also suggests that notch signaling can enhance myelination in a ligand-dependent manner (Hu et al., 2003).

**Figure 1:**
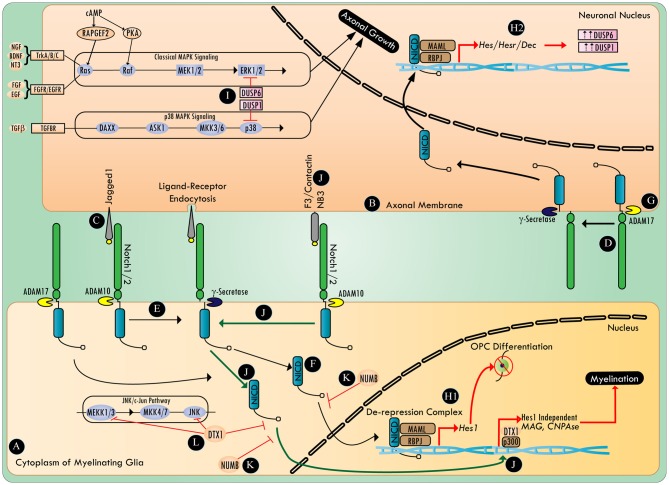
**The involvement of Notch in the regulation of myelination and axonal growth.** Myelinating glia **(A)** and the axonal membrane **(B)** both harbor notch ligands **(C)** and notch receptors **(D)**. Proteases (e.g., ADAM10 and γ-secretase) activate the canonical notch signaling pathway **(E)** by processing the ligand bound notch receptor to release notch intracellular domain (NICD) **(F)**. The protease ADAM 17 can activate canonical notch signaling in a ligand independent manner **(G)**. NICD undergoes translocation to the nucleus and binds to a transcriptional de-repression complex containing RBPJ/MAML transcription factors **(H1,H2)**. In myelinating glia, canonical notch signaling activates a cascade of downstream genes (e.g., *Hes1*), halting OPC differentiation **(H1)**. Whereas, in neurons, canonical notch signaling upregulates DUSP1 and DUSP6 in a *Hes/Hesr/Dec* dependent manner **(H2)**. DUSP1 and DUSP6 inhibit axonal growth and neuritogenesis by negatively regulating the p38 MAPK and ERK_1/2_ pathways, respectively **(I)**. Novel notch ligands (e.g., F3/contactin or NB3) activate non-canonical notch signaling **(J)** that recruits the NICD/Deltex1 (DTX1)/p300 transcription complex, thus activating genes that are essential for myelination. The notch antagonist, numb, inhibits various steps in both the canonical and non-canonical notch signaling pathway **(K)**, whereas DTX1 plays a critical regulatory role in both non-canonical notch signaling and the c-Jun N-terminal kinase (JNK) signaling pathway **(L)**.

Notch signaling (Figure 1) begins with trans-binding of the ligands to notch receptors. This event leads to proteolytic cleavage of the notch extracellular truncated domain, first by a disintegrin and metalloprotease (ADAM), and then by γ-secretase, which releases the notch intracellular domain (NICD) to permit its translocation to the nucleus (Andersson et al., 2011). In the nucleus, in conjunction with the RBPJ/MAML (recombining binding protein suppressor of hairless/ mastermind-like) transcription activation complex, NICD acts to de-repress notch target genes such as *Hes/Hesr/Dec* (hairy/enhancer; hairy/enhancer related; differentiated embryo chondrocyte 1; Andersson et al., 2011).

Hu et al. (2003) proposed a switch model in which axonal jagged-1 expression initially blocks OPC differentiation in a *Hes1* dependent manner. Then, as jagged1 expression decreases with development, the interaction of F3/contactin with notch1, notch2 or NB3 with notch1, could then promote nuclear translocation of NICD, and the transcription of myelin-associated genes, myelin-associated glycoprotein (MAG) and CNPase, in a RBPJ/*Hes1* independent manner (Wang et al., 1998; Hu et al., 2003; Popko, 2003; Cui et al., 2004). This non-canonical notch activation was found to be exclusively dependent on F3/Contactin/NB3, and was mediated by Deltex1 (DTX1), an E3 ligase also known to antagonize c-Jun N-terminal kinase (JNK), and promote the degradation of NICD, as well as MAPK/ERK kinase kinase 1 (MEKK1; Liu and Lai, 2005; Zhang P. et al., 2010). However, given the role of DTX1/p300 in functioning as a non-canonical downstream transcriptional regulator of notch, the exact mechanism of transcriptional activation of myelin-associated genes by DTX1 remains to be elucidated (Yamamoto N. et al., 2001). It is plausible that parallel activation of other axon-glia signaling might post-translationally influence the activity and interactions of DTX1, and direct it towards promyelination signaling. In an apotransferin-induced-cortical remyelination model, notch activation correlated with F3/Contactin expression (Aparicio et al., 2013). Increasing expression of F3/contactin and NB3 during the early postnatal period has been documented in the rat spinal cord (Cui et al., 2004). This apparent change of ligands for the same receptor (notch1) promotes maturation of OLs on demand from axons (Givogri et al., 2002; Hu et al., 2003). In such a scenario, if axons continue to maintain a high expression of jagged1, they can potentially tip the balance of myelination signals towards its inhibition.

Among the 30 members of the ADAM family of proteolytic enzymes, ADAM10 and ADAM17 play an important role in myelination within the PNS (van Tetering et al., 2009; La Marca et al., 2011; Palazuelos et al., 2014). ADAM10 cleaves notch1 in a ligand-dependent manner, whereas cleavage of notch by ADAM17 is ligand-independent (Bozkulak and Weinmaster, 2009). In addition, cytoplasmic notch activity is known to be negatively regulated by numb (Puca and Brou, 2014). The exact mechanism of numb-mediated negative regulation of notch signaling is not known, but multiple mechanisms are proposed, including its interference with NICD endocytosis, NICD nuclear translocation, and notch/RBPJ/MAML-mediated transcription of genes (Giebel and Wodarz, 2012; Flores et al., 2014).

Notch1 is also required for the timely differentiation of neuronal progenitors, and cells that are deficient in notch1 undergo premature neurogenesis, but die by apoptosis before completing the terminal differentiation into post-mitotic neurons (Lutolf et al., 2002). Expression of notch1 in 6 DIV (day *in vitro*) mouse E16–18 cortical neurons, and neurite forming NB2A cells, inhibits neurite extension (Berezovska et al., 1999; Franklin et al., 1999). Notch signaling was also demonstrated to inhibit axonal regeneration in C.*elegans* after axotomy, and preventing notch activation post-injury resulted in enhanced regeneration (El Bejjani and Hammarlund, 2012). Further, numb was shown to reverse notch-mediated axon growth inhibition in 6 DIV cultures of E16–18 mouse cortical neurons, highlighting the importance of negatively regulating notch signaling to promote neuritogenesis (Berezovska et al., 1999; Puca and Brou, 2014). In mice, subsequent to compressive SCI, the expression of numb was observed to be predominantly upregulated in both NeuN-positive neurons and GFAP-positive astrocytes, in rostral as well as caudal spinal cord, for up to 10 mm from the lesion (Chen et al., 2005; Wilhelmsson et al., 2012). However, following injury to the nervous system, output of the notch signaling pathway with the effects of this increased expression of numb remains unknown.

In a mouse SCI compression model, notch1 expression significantly increased both at the mRNA and protein level (Yamamoto S. et al., 2001; Chen et al., 2005). In contrast to numb, notch1 mRNA expression was identified for up to 10 mm rostral and caudal to the injury, exclusively within neurons (Chen et al., 2005). Notch1 mRNA expression was detected beginning 2 days post injury and was still apparent at 14 days post injury (Chen et al., 2005). Notch-mediated inhibition of axonal regeneration appears to be ligand-independent, a finding that could potentially explain the absence of an improvement in hind limb motor function when the jagged1 antagonist (Jagged1-Fc-Chimera) was administered intravenously in mice immediately following a 50 kdyn thoracic T10 contusion SCI (Fassbender et al., 2011; El Bejjani and Hammarlund, 2012). Interestingly, inactivation of γ-secretase using its inhibitor, DAPT (N-[N-(3,5-difluorophenacetyl)-L-alanyl]-S-phenylglycine-t-butyl ester), was sufficient to overcome notch-mediated axonal growth inhibition in C.*elegans* following LASER-assisted axotomy (El Bejjani and Hammarlund, 2012). However, DAPT, which eventually decreases the available NICD pool, was ineffective when applied 2 h after LASER-assisted axotomy, suggesting an alteration in the post-injury molecular milieu, that could provide resistance to notch modulation through putative transcriptional events and post-translational modifications.

The signaling cascade involved in axon growth inhibition via notch activation needs further elucidation (Figure 1). In a RBPJ dependent manner, notch signaling has been shown to lead to the dephosphorylation of ERK_1/2_, and the inhibition of the Ras/Raf/MKK_1/2_/ERK_1/2_ pathway through the upregulation of MAPK phosphatase (Lip-1/MKP-3/DUSP6), and the antagonism of p38 MAPK by the up-regulation of MKP-1 (DUSP1; Muda et al., 1996; Berset et al., 2001; Kondoh et al., 2007). Notch signaling can regulate p38 MAPK/JNK by positively regulating the mTOR pathway in a c-myc dependent manner to increase the expression of MKP1 (DUSP1) via Akt (Protein Kinase B; PKB) signaling (Chan et al., 2007; Rastogi et al., 2013). The role of the p38 MAPK pathway and the downstream effector of the JNK pathway, c-jun, are now being recognized as the critical molecules required for resetting SC fate towards a reparative phenotype (Arthur-Farraj et al., 2012; Yang et al., 2012). The p38 MAPK and JNK pathways are also specifically important in axonal regeneration (Nix et al., 2011). However, work in C.*elegans* has demonstrated that notch can negatively influence axonal regeneration without affecting the DLK-1/MEK_4–7_/JNK pathway (El Bejjani and Hammarlund, 2012). Reports have also suggested that there are cross talks between NICD and the canonical β-catenin pathway, as well as NF-κB, HIF1, and TGFβ-BMP signaling pathways (Andersson et al., 2011; Bonini et al., 2011). Hence, notch mediated down regulation of axonal growth could primarily be due to its negative regulation of the p38 MAPK and ERK pathways, and its effects on myelination could be due to a complex modulation of the transcriptional network involved with myelin-associated gene expression and its indirect modulation of Akt signaling (Flores et al., 2008).

Conversely, multiple reports have highlighted that there is post-translational regulation of NICD by MAPK/ERK, which influences the transcriptional output of canonical notch signaling (Stockhausen et al., 2005; Tremblay et al., 2013; Yamashita et al., 2013). In addition, the MAPK signaling pathway shares multiple substrates with the cyclic AMP/PKA cascade and in turn can be regulated by cyclic AMP in a PKA-dependent (PKA/RhoA or PKA/PTP) or PKA-independent (Epac/Rap1 or Epac/Rit) manner (Gerits et al., 2008). Anecdotal reports of cyclic AMP being able to upregulate jagged 1 levels in osteoblasts, in a PKA-dependent manner, supports the potential existence of a wider notch regulatory network in neural cells (Weber et al., 2006). Examining the cross talk between notch signaling and cyclic AMP after injury, under conditions where levels of cyclic AMP are dramatically reduced (Pearse et al., 2004; Hannila and Filbin, 2008; Lau et al., 2013), may reveal novel players at the intersection of those signaling pathways.

In summary, notch signaling (Figure 1) constitutes an emerging component of axon-glia communication during injury. Notch interaction with its ligands plays an important role in modulating myelination, and warrants further work to better define these relationships, and to identify the intermediaries involved in these processes. In particular, nodes involving the interaction of notch with MAPK signaling (e.g., DUSPs) and cytoskeletal network may offer unique therapeutic targets for enhancing remyelination repair and axonal regeneration.

### Neuregulin-ErbB Signaling

**Figure 2:**
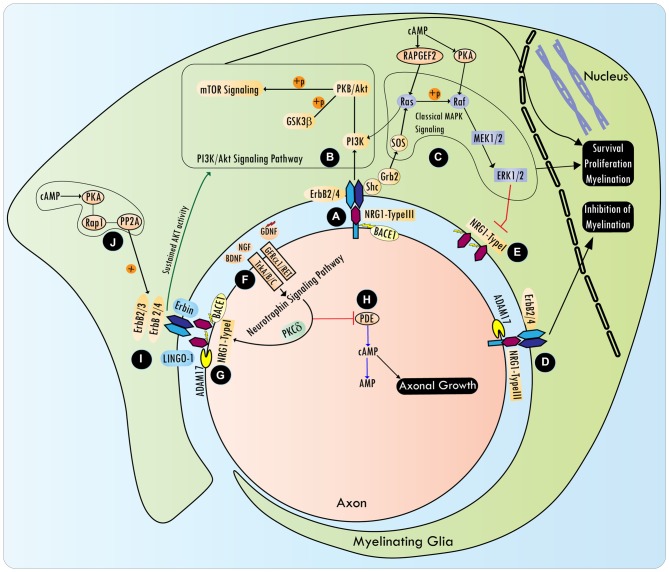
**NRG/Erb signaling in the control of peripheral myelination and axonal growth.** BACE1 processed axonal NRG1-type III interacts with ErbB receptors **(A)**, and promotes cell survival, proliferation and myelination by activating various signaling pathways, including mTOR via PI3K/Akt **(B)** and ERK_1/2_ via Ras/Raf **(C)**. However, NRG1 type III cleavage by ADAM17 conveys inhibitory signals to the myelination program **(D)**. In Schwann cells (SCs), axonal NRG type III (transmembrane) represses NRG type I (soluble) isoform expression via ERK_1/2_ **(E)**. Rapid axonal NRG1 release can be spatially regulated by neurotrophic factors [e.g., Nerve growth factor (NGF), brain-derived neurotrophic factor (BDNF), and glia cell-derived neurotrophic factor (GDNF)], that are released by SCs, and act via TrkA, TrkB or GFRα1/RET receptors, respectively, on axonal surfaces **(F)**. Neurotrophins induce the coordinated efforts of PKCδ and cell surface proteases such as BACE1 and ADAM, which cleave a precursor form of axonal NRG1 to release its active soluble form **(G)**. In addition, neurotrophins also activate the Shc/PI3K/Akt survival pathway and Shc/Ras/ERK_1/2_ differentiation pathways. Activation of ERK by neurotrophins can inhibit PDE4 (phosphodiesterase) activity, which leads to the antagonism of cyclic AMP hydrolysis **(H)**. Elevating intracellular cyclic AMP is critical to overcoming axon growth inhibition by myelin-associated molecules, such as myelin-associated glycoprotein (MAG), which is present in non-compact myelin. NRG1/ErbB signaling is further regulated by the adaptor protein Erbin and ErbB2 spatial localization determinant protein, LINGO-1, a component of the MAI-Nogo receptor-signaling complex **(I)**. Cyclic AMP can sustain NRG1 type I-mediated transient Akt phosphorylation via PKA dependent phosphorylation of the ErbB receptor, a mechanism that involves Rap1b-PP2A signaling **(J)**. NRG1/ErbB signaling is dispensable for the myelination program in the CNS, and a detailed understanding of the NRG1/ErbB interaction network following the CNS injury is lacking.

NRG1 is a member of the neuronal growth and differentiation factor family best known to be critical for SC development (Birchmeier and Nave, 2008). NRG1, the most studied of the four neuregulin genes, produces at least 15 different isoforms from multiple transcription start sites and alternative splicing (Nave and Trapp, 2008). All the six main isoforms of NRG1: type I (Heregulin: HRG; soluble), II (glial growth factor: GGF), III [sensory motor neuron-derived factor (SMDF); transmembrane], IV, V and VI have a similar epidermal growth factor (EGF)-like domain but distinct N-terminal regions. Expression of NRG1 has been detected in the uninjured spinal cord, as well as following SCI, in neurons, axons and OLs (Vartanian et al., 1999; Gauthier et al., 2013). NRG1 binds to membrane spanning receptor tyrosine kinases (RTKs), ErbB3 and ErbB4, which are part of the EGF receptor superfamily (Iwakura and Nawa, 2013). CNS expression of ErbB3, and to some extent that of ErbB4, is observed exclusively in OLs (Sussman et al., 2005; Makinodan et al., 2012). ErbB receptor subunit expression is present in the adult brain, spinal cord as well as dorsal root ganglia (DRGs; Bermingham-McDonogh et al., 1996; Martínez et al., 2004; Pearson and Carroll, 2004). In the PNS, SCs predominantly express ErbB2 and ErbB3 (Garratt et al., 2000). Binding of NRG1 to ErbB3 or ErbB4 leads to the activation of multiple signaling pathways (Figure 2), ensuing heterodimerization with ErbB2, since ErbB3, which contains a pseudokinase domain, cannot activate downstream effectors (Weiss et al., 1997; Maurel and Salzer, 2000; Burgess et al., 2003). Proteolytic cleavage of the NRG1 N-terminal domain by ADAM17 or β-secretase, is a prerequisite for NRG1/ErbB2/3 signaling (Ronchi et al., 2015).

Much of our understanding regarding the role of NRG1 in the process of myelination comes from experiments with knockout mice (Brinkmann et al., 2008; Newbern and Birchmeier, 2010). Conditional knockout of NRG1 in cortical projection neurons, before the onset of cortical myelination, did not lead to any changes in the myelin assembly of the subcortical white matter or spinal cord, whereas parallel observations within CNS-PNS border zones suggested that SC development and its myelination program were altered (Dragatsis and Zeitlin, 2000; Michailov et al., 2004; Brinkmann et al., 2008). In nestin-cre driven NRG1 knockout mice, changes in the CNS were again largely unremarkable, though animals did exhibit early lethality (Brinkmann et al., 2008). However, in spinal cord explants obtained from the NRG1 knockout mice, a selective and severe reduction in OL development was observed that could be rescued with recombinant NRG1 (Vartanian et al., 1999). Conversely, when NRG1 type I or type III were overexpressed under a Thy1.2 driver in mice, hypermyelination was seen in thin (0.4 μm) neocortical fibers, without an overt change in OL numbers (Brinkmann et al., 2008). In the same study, NRG1 type III overexpression lead to premature myelination in the mouse optic nerve (Brinkmann et al., 2008). Closer examination of the OLs in the optic nerve of these mice showed that they exhibited an increase in soma size and a widened territory of coverage by their processes, suggesting that NRG1 overexpression could produce subtle changes in OL morphology (Brinkmann et al., 2008). OLs have been shown to respond in culture to soluble NRG1 by producing galactocerebroside and myelin basic protein (MBP; Vartanian et al., 1997, 1999; Fernandez et al., 2000; Calaora et al., 2001). Thus, discrepancies between *in vivo* and cell culture observations with OLs in NRG1 knockout animals highlight some limitations of the models used, though they do suggest that NRG1 may play a role in OL function and myelination at later stages, but it is largely dispensable for myelination during CNS development.

On the contrary, loss of ErbB2 produces a severe reduction in OL numbers, as well as an impairment in their axon ensheathing capability (Park et al., 2001). The development of a transgenic mouse in which a dominant negative ErbB2 was expressed specifically in OLs, through use of a MBP promoter, showed that competitive antagonism of ErbB2 resulted in widespread hypomyelination and defects in OL differentiation (Kim et al., 2003). Similarly, the prevention of ErbB2 translocation to lipid rafts by LINGO-1, a component of the MAI-Nogo receptor-signaling complex, prevents OL differentiation (Lee et al., 2014). In the PNS, effective NRG1-ErbB myelination signaling needs Erbin expression in SCs, a leucine rich repeat (LRR) and PDZ domain-containing adapter protein belonging to the LAP family that interacts with ErbB2 (Borg et al., 2000; Tao et al., 2009). Erbin also appears to be an essential component for peripheral axon remyelination after injury (Liang et al., 2012). Interfering with Erbin expression inhibits NRG1 mediated Akt activation (Tao et al., 2009). These findings collectively suggest an important role of ErbB2, not only in the maturation of myelinating glia during development, but also for their functioning in repair when the nervous system is injured. In the CNS, ErbB3 knockout, in contrast, has no effect on OL differentiation and myelination (Schmucker et al., 2003). OLs from mice with an OL specific knockout of ErbB3 and ErbB4 were still observed to myelinate the CNS in a timely manner as compared to their wild type controls (Brinkmann et al., 2008). On the contrary, activation of ErbB3 leads to OL proliferation and differentiation, whereas ErbB4 activation leads to the suppression of OL maturation (Sussman et al., 2005; Makinodan et al., 2012). Further studies with the selective targeting of ErbB receptors during injury remain to be undertaken.

Neurotrophic factors secreted by SCs can spatially regulate rapid axonal NRG1 release (Esper and Loeb, 2004). Nerve growth factor (NGF), brain-derived neurotrophic factor (BDNF) and glia cell-derived neurotrophic factor (GDNF) released by SCs act via TrkA, TrkB or GFRα1/RET receptors respectively, on axonal surfaces. These neurotrophins provide trophic support to underlying axons and drive NRG1/ErbB signaling in SCs within the vicinity of axons, as well as regulate their myelination ability (Hoke et al., 2003; Ascano et al., 2009; Esper and Loeb, 2009). Neurotrophins induce the coordinated actions of PKCδ and cell surface proteases such as BACE1 and ADAM, which cleave a precursor form of axonal NRG1 to release its active soluble form (Loeb et al., 1998; Esper and Loeb, 2009). In addition to activating PKC via the PLCγ pathway, neurotrophins activate the Shc/PI3K/Akt survival and Shc/Ras/ERK_1/2_ differentiation pathways (Ohira and Hayashi, 2009). Activation of ERK by neurotrophins can inhibit PDE4 (phosphodiesterase 4) activity, which leads to the antagonism of cyclic AMP hydrolysis (Figure 2). Elevating intracellular cyclic AMP is critical to overcoming axon growth inhibition by myelin-associated molecules, such as MAG, which are present in non-compact myelin (Gao et al., 2003; Patzig et al., 2011). NGF differentially regulates myelination in the CNS and the PNS, inhibiting myelination by OLs, while promoting myelination by SCs (Chan et al., 2004). Though work remains to improve our understanding of the modulation NRG1/ErbB signaling by neurotrophins in OLs, this signaling interaction has important implications for transplantation experiments after SCI that involve the use of genetically modified SCs with the overexpression of neurotrophins.

Axonal NRG1 interacts with ErbB2/3 to activate various signaling pathways (Figure 2; Ras/ERK_1/2_, NF-κB, Ras/PI3K/Akt, Shc/Ras/PI3K, Shc/Shp2/Src/FAK and PLCγ/Calcineurin) that promote cell survival, proliferation and myelination (Newbern and Birchmeier, 2010; Heermann and Schwab, 2013). PI3K/Akt signaling downstream of NRG1/ErbB can serve as an additional mechanism to promote myelination, even at later stages of the development (Flores et al., 2008; Goebbels et al., 2010). Cyclic AMP, when used as a mitogen in SC cultures, can sustain NRG1 type I (heregulin)-mediated Akt phosphorylation via PKA dependent phosphorylation of the ErbB receptor, a mechanism that involves Rap1b-PP2A signaling (Monje et al., 2006; Hong et al., 2008). Conversely, constitutively active Akt (possibly acting through mTOR) can enhance myelination in the CNS without affecting OL numbers, but does not seem to have any role in the PNS myelination (Flores et al., 2008; Narayanan et al., 2009). The NRG1/ErbB signaling pathway also has an extensive cross talk (covered below) with other signaling pathways originating from notch, neuronal merlin, β integrins and GPR126/cyclic AMP adhesion G protein (Pietri et al., 2004; Woodhoo et al., 2009; Mogha et al., 2013; Schulz et al., 2014; Petersen et al., 2015). Recently, a nuclear variant of ErbB3 has been identified, which is under transcriptional control of NRG1; siRNA knockdown of nuclear-ErbB3 in SC-neuron co-cultures lead to a nearly 50% reduction in myelin segments, prompting a re-evaluation of the role of ErbB3 in myelination (Adilakshmi et al., 2011).

The current understanding of the involvement of NRG in peripheral regeneration following injury is that the axonal NRG type III (transmembrane) represses NRG type I (soluble) isoform expression in SCs via ERK_1/2_ (Stassart et al., 2013). After the injury, the autocrine/paracrine NRG1 signals originating from SCs take over to promote de-differentiation and myelination by denervated SCs (Raphael et al., 2011; Stassart et al., 2013; Mei and Nave, 2014). However, studies in peripheral nerve injury also provide evidence that following injury, axonal NRG1, though not essential, is required for remyelination in a rate limiting fashion (Fricker et al., 2009, 2011, 2013).

Following injury, in the PNS, NRG1/ErbB isoforms show a differential expression pattern during the degeneration and regeneration phases (Ronchi et al., 2015). Intriguingly, NRG1 type III (b/c) is upregulated in the regeneration phase of peripheral nerve injury via a reversible switch between ADAM17 to BACE1 dependent cleavage (Ronchi et al., 2015). ADAM17, a ligand-independent activator of notch signaling, has previously been shown to produce NRG1 type III cleavage that is inhibitory to SC myelination (Bozkulak and Weinmaster, 2009; La Marca et al., 2011). BACE1, on the other hand, is known to produce a promyelinating cleavage product of NRG1 type III, but this product was recently deemed non-essential for the peripheral myelination program (Willem et al., 2006; Velanac et al., 2012). These studies highlight the dichotomy and dynamic nature of decision making between myelination and axon growth programs in the PNS. Whether similar mechanisms operate following injury to the CNS is currently unknown. Following injury, ErbB2 protein levels are upregulated by 3rd day and persist for 4 weeks, whereas ErbB3 expression is upregulated at around 7 days and persists until 4 weeks (Ronchi et al., 2015). Intriguingly, the protein level of ErbB2 does not reflect the pattern of mRNA expression, which is downregulated after PNS injury. This can be related to a relative resistance of ErbB2 to degradation by several debated mechanisms that include efficient recycling, regulation of its endocytosis and HSP90/Cdc37 induced stabilization, or post-transcriptional regulation by the ERK/PDE/cAMP/PKA network (Bertelsen and Stang, 2014). Together, these studies suggest that NRG/ErbB signaling could be regulated in a complex manner, especially after injury, and more work needs to be done to iron out the discrepancies that exists among various studies.

Within 1 day following moderate thoracic contusion SCI in mice with the OSU device, NRG1 type I expression increases at the lesion site, which after 2 weeks, returns to control levels (Lasiene, 2009). Conversely, the expression of NRG1 type III was found to remain significantly decreased at all time points following the injury (Lasiene, 2009). In the same study, intrathecal infusion of NRG1 type III but not NRG1type I-β1 (Ser2-Lys246) into the mouse spinal cord lead to a transient increase in myelin sheath thickness in the axons caudal to the injury site (Lasiene, 2009). On the contrary, a study using a compressive thoracic SCI model in rats observed a downregulation of NRG1 type I expression following injury, along with no change in ErbB2/3/4 receptor expression (Gauthier et al., 2013). In a similar SCI model (compression) sustained intrathecal infusion of NRG1 type I-β1 (Thr176-Lys246) to the spinal cord was able to increase the expression of CNPase (a marker of OLs) and NF200 (axonal neurofilament), which was reversed by an ErbB2/4 inhibitor (Gauthier et al., 2013). In addition, a study administering subcutaneous NRG1 (soluble Type II; GGF2; Nrg1-β3) 24 h after thoracic contusion SCI in rats (weight drop, 10g/2.5 cm height) and mice (60 kdyn, T9), for a delivery period of 7 days, showed an increase in adult OL number and a subsequent increase in the myelination of spared axons (Whittaker et al., 2012). These findings highlight the differential characteristics of NRG1 isoforms. In extrapolating the usefulness of different NRG1 isoforms for therapeutic purposes, it is important therefore that NRG1 domains and amino acid stretches are adequately identified, in addition to the SCI model system used for a research study (Cheriyan et al., 2014). It is likely that some amino acid stretches on NRG1 domains might interact with pathways countering OL survival and myelination programs. A detailed evaluation of this research direction remains to be undertaken.

Studies to date suggest that the requirement of NRG1 *in vivo* is different for the PNS and the CNS. In the PNS, NRG1 is essential for SC differentiation and myelination, however, for the CNS, even though NRG1 is capable of altering the myelination process, it is dispensable (Brinkmann et al., 2008). However, after the injury there is considerable evidence that NRG1/ErbB signaling might play an important role in re-myelination and the survival of glia in both the CNS and the PNS (Lasiene, 2009; Whittaker et al., 2012; Gauthier et al., 2013). Even though there is anecdotal *in vitro* evidence that NRG1-β1 can promote neurite outgrowth, a majority of studies report that NRG1 promotes neuronal survival and acts as a neuroprotectant in adult injured tissues (Bermingham-McDonogh et al., 1996; Zhang et al., 2004; Edwards and Bottenstein, 2006; Iaci et al., 2010; Li et al., 2012; Whittaker et al., 2012). Accumulating evidence suggests that different isoforms of NRG1 may hold the key to understanding their precise role in CNS myelination and axon regeneration following injury. Specifically, there is a need to revisit the role of NRG1 isoforms, and the signaling cascade emanating from lipid-raft-inserted ErbB2 for its role in modulating the myelination program. Emerging understanding of NRG1/ErbB signaling necessitates further experiments to determine its broader clinical significance.

### β-Secretase and Metalloprotease Signaling

Neuronally expressed β-site amyloid precursor protein (APP) cleaving enzyme1 (BACE1), a transmembrane protein and an asparatyl protease that is upregulated during PNS myelination, is another player in NRG1/ErbB signaling (Willem et al., 2006). BACE1, as well as ADAM10 and 17, can cleave NRG1 (type I and type III) at their C-termini to their EGF domains, whereas BACE1 and ADAM17 can also cleave NRG1 type III at its N-terminus to its EGF domain, releasing them from the neuronal membrane and assisting in their paracrine signaling (Montero et al., 2000; Syed and Kim, 2010; Luo et al., 2011; Fleck et al., 2013). NRG1 N-terminal cleavage releases α-sEGF and β-sEGF by ADAM17 and BACE1 respectively, whereas C-terminal cleavage by ADAM or BACE1 releases α/β-CTF, which undergoes rapid turnover (Fleck et al., 2013). Both α/β-sEGF can activate ErbB3 phosphorylation and promote downstream Akt signaling (Fleck et al., 2013). Crude N-terminal fragment of NRG1 (NTF) activates Akt in a similar fashion (Luo et al., 2011). However, BACE1-mediated processing of NRG1 was found to be crucial for myelination in SC-DRG co-culture studies (Luo et al., 2011). BACE1 released β-sEGF was sufficient to rescue peripheral hypomyelination in BACE1 mutant zebrafish (Fleck et al., 2013). Recently, for the first time, a study observed NRG1 cleavage to switch between two enzymes (ADAM17 to BACE1 and back to ADAM17) over the course of regeneration, after rat sciatic nerve crush as well as in a neurotmesis repair paradigm (Ronchi et al., 2015).

BACE1 knockout mice show a specific defect in myelination and not in axonal ensheathment. This is exemplified by the decreased expression of MBP and PLP proteins, enriched in OL-laid compact myelin, and normal expression levels of MAG, a protein enriched in the periaxonal membrane, in BACE1 knockout mice (Hu X. et al., 2006). Expectedly, in BACE1 knockout mice, full length NRG1 was increased and a reduced activation of PI3K-Akt was noted (Hu X. et al., 2006). However, a re-evaluation of the BACE1 knockout showed more specific defects in remyelination following cuprizone induced demyelination (Treiber et al., 2012). CNS hypomyelination in BACE1 knockout mice was observed previously in hippocampal and optic nerves, while work by Treiber et al. (2012), in the corpus callosum, suggested region specific differences in the myelination program in the brain, as observed in other studies (Tomassy et al., 2014). Recently, breeding BACE1 knockout mice to mice expressing a constitutively active Akt (Akt-DD), specifically in OLs, showed rescue of BACE1-induced CNS hypomyelination by Akt, reinforcing that NRG1-ErbB/Akt signaling is downstream of BACE1 (Hu et al., 2013). In the PNS, BACE1 was present on both axons and SCs, and identified as important for the proper myelination of axons (Fleck et al., 2012; Hu et al., 2015).

Understanding the substrates of BACE1 during different phases of regeneration can provide unique insights into the PNS regeneration program. Studies have indeed applied quantitative proteomics to identify the substrates of BACE1 in cell models and zebrafish (Hemming et al., 2009; Hogl et al., 2013). At least 24 unique proteins accumulate in the membrane fractions of brain from BACE1 knockout zebrafish, suggesting them to be putative substrates (Hogl et al., 2013). Many of the molecules are involved in axonal growth, guidance and sprouting such as NCAM, L1, Plexin A3 and Glypican1 (Jakeman et al., 2006; Zhang et al., 2008; Bai and Pfaff, 2011; Shen, 2014). These findings stress the need for further work to understand the importance of BACE1 specific substrates in clinically related injury models of the PNS and CNS.

### Fibroblast Growth Factor-2 (FGF2) Signaling

FGF2 isoforms have been known to be differentially regulated in the DRG and sciatic nerve (Meisinger and Grothe, 1997). DRG neurons express both FGFR1 and FGFR2 (Grothe and Nikkhah, 2001; Hausott et al., 2011). OPCs and differentiated OLs express FGF receptors in a developmentally-regulated manner. OPCs express FGFR1 and FGFR3, and differentiated OLs express FGFR1. On the other hand, paranodal myelin shows clusters of FGFR2 on lipid rafts of OLs (Bansal, 2002; Bryant et al., 2009). Downstream signaling for FGFR1/2 seems to occur via Raf-MEK-ERK_1/2_ and PI3K/Akt/mTOR pathways, but it is plausible that FGF1/2 signals are also transmitted by other receptors (β1 integrins), and affected via other various intracellular signaling pathways (Grothe and Nikkhah, 2001; Hausott et al., 2011; Ornitz and Itoh, 2015).

Various FGF isoforms exert differential effects on OLs and OPCs. Specifically, FGF-2 promotes proliferation and inhibits differentiation of OPCs *in vitro*, but promotes process elongation, cell cycle re-entry, and decreases MBP protein expression in mature OLs (Fortin et al., 2005). Even though FGF2 signaling for OPC proliferation *in vivo* and *ex vivo* seems to be dispensable, a decrease in MOG, MBP transcription, and thickness of the myelin sheath have been observed after the long term absence of both FGFR1/2 in OLs (Furusho et al., 2012). On the contrary, administration of FGF2 to SCs in culture decreases the expression of P0 mRNA as well as its protein. In addition, FGF2 was able to inhibit the positive regulation of the myelin sheath component protein P0 via Forskolin, an activator of adenylyl cyclase (Morgan et al., 1994). These studies highlight the differential effect of FGF signaling in CNS and PNS glia, and point towards co-regulators that could be driving such a differential response.

Intrathecal injection of bFGF (30 min to 1 h post spinal contusion injury, infusion for 7 days), has been shown to produce positive effects on tissue preservation (Lee et al., 1999; Rabchevsky et al., 1999). Recently, acute subcutaneous administration (within 30 min of injury, then every 2 days post SCI) of human FGF2 was carried out in a rat spinal cord hemisection injury model showing decreased gliosis and a concurrent decrease in chondroitin sulfate proteoglycans (CSPGs; Goldshmit et al., 2014). Additionally, increased neuronal progenitor and radial glial numbers, as well as a change in the morphology of the glial scar and glial cell morphology (bipolar), towards that are supportive for axonal regeneration, were observed with FGF2 (Goldshmit et al., 2014). Combinatory treatment of a spinal thoracic transection injury in rats with a SC-fibrin bridge, along with recombinant human FGF2, produced a 3–4 fold increase in surviving NeuN positive cells in the adjacent host cord as compared to control (Meijs et al., 2004). However, recombinant human FGF2 failed to produce any significant growth of axons into the bridge, and did not lead to improved functional recovery (Meijs et al., 2004). By overexpressing the different isoforms of FGF2 in SCs and using them in a sciatic nerve graft, Haastert et al. (2006) demonstrated that FGF2–21/23 kDa (High Molecule Weight FGF2; HMW-FGF2) isoform preferentially promoted myelination whereas FGF2–18 kDa (Light Molecule Weight FGF2; LMW-FGF2) isoform was observed to be inhibitory to the myelination of regenerated axons (Haastert et al., 2006). In addition, the studies suggested that LMW and HMW FGF2 differentially regulated sensory and motor neuron regeneration and functional recovery (Haastert et al., 2006; Allodi et al., 2013). Other studies have shown that SCs overexpressing FGF2 enhance peripheral nerve regeneration (Danielsen et al., 1988; Fujimoto et al., 1997; Timmer et al., 2003). It is plausible, however, that FGF2 has pleotropic effects on myelination and axonal regeneration. Studies in FGF2 knockout mice support this view; where following sciatic nerve crush injury, distal to the crush site, at least 5× the number of regenerating axons were present as compared to control. This improvement is seen along with increased myelination and axon diameter, as well as enhanced sensory recovery, which the authors ascribed to an enhanced myelin clearance (Jungnickel et al., 2004, 2010).

In summary, these findings support that there exists a differential, isoform specific role of FGF2 in axon regeneration. Further experiments are required to understand the role of FGF2 isoforms and the modulation of FGFR endocytosis in relation to axonal regeneration and myelination following SCI (Goldshmit et al., 2012; Adeeb and Mortazavi, 2014).

### Insulin Growth Factor (IGF) Signaling

IGF-I and II are polypeptides that play an important role in the development and maturation of neurons and glia, particularly projection neurons and cerebellar neurons (Andersson et al., 1988; Liu et al., 2000). Neurons secrete IGF-1 during activity via somatodendritic exocytosis in a syaptotagmin-10 dependent manner (Cao et al., 2011). IGF works through IGF receptor 1 and 2 (IGF1R, IGF2R), which are expressed on OLs. IGF receptor (IGF1R) abundance decreases with age (Garofalo and Rosen, 1989). IGF-1 signaling seems to be crucial for OPC survival, proliferation, and differentiation as well as CNS myelination (D’Ercole et al., 2002). Similarly in the PNS, IGF-1 promotes survival, proliferation and differentiation of SCs by inducing myelin-associated P0 protein expression (Cheng et al., 1996; Stewart et al., 1996; Cheng and Feldman, 1997; Sondell et al., 1997). In OL-specific IGF-1 knockout mice (Olig1-cre and PLP-cre driver), a severe reduction in CC1^+^ mature OLs as well as NG2+ OLs was observed, along with a decrease in the myelin-associated protein PLP (Zeger et al., 2007). In global IGF-1 knockout mice, cortex, hippocampus and diencephalon show the maximum effect of decreased myelination, whereas brainstem and cerebellum show modest demyelination (Ye et al., 2002). However, post-demyelination, a local increase of IGF1 mRNA levels in the spinal cord failed to produce any OL-mediated myelination (O’Leary et al., 2002). The relevance of IGF-1 signaling with respect to myelination of long axonal tracts in the spinal cord and long relay neurons has yet to be established (Lee et al., 1992; Bibollet-Bahena and Almazan, 2009; De Paula et al., 2014). These findings highlight the need to search for co-modulators of CNS IGF signaling. Interestingly, SCs express IGF-1 and IGF-II, and are the most abundant source of IGF-1 supply in extra-ocular muscles (Kerkhoff et al., 1994; Feng and Von Bartheld, 2010). However, besides IGF1 expression, SCs also express IGF-binding proteins such as IGFBP4 and 5, which modulate IGF1 action. IGFBP4 was shown to inhibit IGF1 action, whereas IGFBP5 works along with IGF1 to promote myelination (Clemmons, 1998; Hammarberg et al., 1998; Cheng et al., 1999a,b).

IGF-1 specifically enhances axonal growth of pyramidal, brainstem and spinal motor neurons (Dobrowolny et al., 2005; Ozdinler and Macklis, 2006). On the contrary, IGF-1 is ineffective for the axonal growth of callosal projection neurons and retinal ganglion neurons (Goldberg et al., 2002; Catapano et al., 2004). Similarly, transplantation of IGF-1 secreting bone marrow stromal cell grafts into a dorsal column hemisection lesion in the rat, led the regeneration of raphespinal and cerulospinal, but not corticospinal axons (Hollis et al., 2009). Together these findings suggest that additional molecules could regulate the downstream response of IGF1 in a cell-specific manner. Recently, the β subunit of IGF1R was observed to be expressed on the distal axons of adult retinal ganglion cells, and negative modulation of IGF1R expression/activity led to nearly 20-fold decrease in axonal regeneration potential of the RGC neurons in culture (Dupraz et al., 2013). Similarly, following transection of the ventral funiculus in the rat lumbar spinal cord, the expression of both IGF-1 and IGFBPs (2, 5 and 6) were found to be upregulated in the ventral and ventrolateral gray matter as well as in the scar tissue (Hammarberg et al., 1998). The combination of the IGF-1 secreting cell graft with the infusion of an IGFBP inhibitory non-peptide ligand (NBI-317712) improved the survival of corticospinal neurons after an internal capsule injury in rats (Hollis et al., 2009). These findings reiterate that further understanding of the downstream bottlenecks of IGF1 signaling could provide novel tools for therapeutic intervention.

Downstream signaling from IGF-1 occurs via the Ras/Raf/MEK/ERK and PI3K/Akt/mTOR pathways. Recently, ubiquitin ligases MDM4/2, along with the transcription factor p53, were observed to form an inhibitory complex that blocked IGF1R signaling (Joshi et al., 2015). Nutilin3, an anticancer drug in development that blocks MDM2-p53 inhibitory interactions, has been shown to enhance axon regeneration in a IGF1R dependent manner (Joshi et al., 2015). Suppressing p53 levels or p53 activity also enhances the reprogramming efficiency of fibroblasts to convert into dopaminergic neurons (Liu et al., 2014; Rasmussen et al., 2014). Together, these findings suggests that in long projection neurons, IGF signaling might be amenable to transcriptional modulation, and calls for further exploration of the mechanisms that increase the expression of IGF1R, decrease the expression of inhibitory IGFBP (thus preventing sequestration of useful IGF) or that enhance IGF1R-mediated signaling.

### Integrin Signaling

Integrins belong to a family of cell surface receptors that recognize ECM proteins such as fibronectin, laminin, collagen, vitreonectin, and which can also bind to other integrin family members (DeSimone et al., 1987; Hynes, 1987). The α and β subunits of integrins come in various forms, and binding of a combined αβ integrin to the ECM promotes a positive feedback loop between integrin clustering, intracellular cytoskeleton assembly and ECM organization (Giancotti and Ruoslahti, 1999). OLs have been observed to express various combinations of αβ integrins, including α_6_β_1_ (Milner et al., 1997). The expression levels of integrins change throughout OPC development and during the generation of mature OLs. In addition, axons themselves considerably affect integrin subunit expression on OLs (Milner and Ffrench-Constant, 1994; Milner et al., 1997). The ligand for α_6_β_1_ integrin, laminin2α, is known to be expressed on retinal projections, dendritic spines, and differentially on myelinating axons of the brainstem and proximal spinal cord (Morissette and Carbonetto, 1995; Tian et al., 1997; Colognato et al., 2002). Laminin is also abundant on the SC basal lamina (Bunge et al., 1986).

Binding of ligand to the β-integrin receptor leads to auto-phosphorylation of focal adhesion kinase (FAK). The FAK/Paxillin complex then recruits the SRC family of protein kinases (Fyn, Src, Lyn and especially Lck) to transfer the signal to CRK/p130^Cas^, which in turn recruits the small GTPase Rac1 to activate NF-κB or JNK pathways (Chen et al., 2000; Schaller, 2001; Iwahara et al., 2004; Ness et al., 2013). In the PNS, Rac1 activated integrin signaling in SCs is crucial for radial sorting and subsequent myelination of PNS axons (Nodari et al., 2007). Binding of ligands to β-integrin can also directly activate the PI3K/Akt survival pathway via FAK.

In a mouse model, the overexpression of dominant negative β1 integrin (dnβ1) reduced FAK activation (p-Tyr_397_) by 43% in optic nerves as compared to controls, but not in the spinal cord, suggesting a region-specific involvement of integrin signaling (Camara et al., 2009). In addition, an increase in the minimum axon diameter required to initiate myelination was noted in the optic nerves of the dnβ1 mice. This is very similar to NRG levels influencing the myelination program (covered below). FAK knockout mice showed similar findings (Forrest et al., 2009). Selective knockout of FAK in OLs, using a PLP-cre driver led to a reduction in the number of myelinated fibers in P14 mouse optic nerves (Forrest et al., 2009). However, at P28, both wild type and mutant dnβ1 and FAK knockout mice, show a comparable number of myelinated axons in the optic nerves, suggesting a transient and region-specific FAK influence. Contrary to the transient nature of the myelination defect in dnβ1 integrin mice, α_6_ integrin knockout mice show hypomyelination due to the apoptotic cell death of OLs (Colognato et al., 2002). Interestingly, OL apoptosis was rescued by NRG1β1 (neuregulin), and OPCs from the α_6_ integrin knockout mouse, when differentiated on laminin2α (α_6_β_1_ integrin ligand) coated plates in the presence of NRG1β1, were able to myelinate appropriately (Colognato et al., 2002). NRG1β1-induced survival and differentiation in the presence of the laminin2α depends on the MAPK signaling pathway, with subsequent phosphorylation (Ser_112_)-mediated inhibition of Bad, a pro-apoptotic molecule, in a PI3K-independent manner (Fang et al., 1999; Colognato et al., 2002). These findings suggested that NRG1β1 was able to switch the dependence of the integrin response from PI3K to MAPK.

β1 integrins interact with the extracellular domain (L1-Fc) of F3/Contactin, a protein enriched on axons that activates Fyn kinase by repressing its inhibitory phosphorylation of Fyn-pTyr_531_ (Laursen et al., 2009). Supporting this finding, OL-specific knockout of contactin-1, using a PLP-cre driver, led to a 46% decrease in OL processes and impaired myelin membrane expansion (Colakoglu et al., 2014). Taken together, these findings implicate an integrated role of integrin-FAK pathway in deciding the onset and delay of myelination, and a cross talk with other molecular pathways could potentially influence its region specificity.

Laminin/β1 integrin signaling assists neuritogenesis by mediating microtubule assembly as well as stabilization in axons (Lei et al., 2012). Recent studies suggest that the integrin ligand, laminin, does this by switching α_3_β_1_/α_7_β_1_ integrin-mediated F-actin dynamics/exocytic signaling from the Ena/VASP/WAVE/VAMP2 pathway to FAK/SRC/Cdc42/Rac/Arp2/3/VAMP7 complex-dependent signaling (Krause et al., 2003; Gupton and Gertler, 2010; Havrylenko et al., 2015). Loss of β1 integrin leads to decreased pLKB1 (Ser431) and SAD-A/B kinase levels, and alters microtubule stabilization via the FAK/SAD pathway (Lei et al., 2012). The mechanistic aspects of LKB1 phosphorylation are elusive. However, PKA might phosphorylate LKB1, since α_4_β_1_ and α_5_β_1_ integrins are known to function as AKAPs (PKA specific A-Kinase anchoring proteins), and Ser431 in LKB1 is a PKA consensus phosphorylation site (Lim et al., 2008; Lei et al., 2012). These findings suggest that integrin-associated Src kinases can integrate signals from axons as well as the basal lamina and interact with a larger network of partners intracellularly.

MAIs further regulate these cell-specific integrin pathways. MAG stimulates asymmetric clathrin and calcium-mediated endocytosis of β1 integrins at the growth cone, and MAG likely performs a similar function elsewhere in axons (Hines et al., 2010). Since MAG is usually present at the axon-glial junction, it is plausible that the dynamic regulation of the surface expression of MAG couples integrin signaling with the myelination program at the axon-glial junction.

In summary, integrin signaling has a profound influence on both myelination and axon regeneration. Axons and glia have been demonstrated to mutually activate and modulate the integrin signaling pathway (Eva et al., 2012; Eva and Fawcett, 2014).

### Cell Adhesion Molecules (CAMs)

CAMs upon the plasma membrane are critical to neuron-glia interactions, bringing neuronal (in this case axonal) and oligodendrocyte membranes together. CAMs either activate signaling pathways themselves or help juxtapose signaling complexes (Pollerberg et al., 2013). In addition, CAMs play an important role in the patterning of axonal functional domains (e.g., node of Ranvier, paranode, juxtaparanodes) with the involvement of adjacent glia (Normand and Rasband, 2015). There are at least four groups of CAMs described, which differ largely in their functional requirement for calcium. Of these, classic cadherins are calcium-dependent cell-to-CAMs that work in tandem with catenins (α, β and p120), their cytoplasmic binding partners, which connect them to the actin cytoskeleton (Takeichi, 2007).

In the nervous system, OLs, SCs and axons express the prototype molecule, N-cadherin, in an age-dependent manner. When N-cadherin function was blocked by a peptide, only 50% of Purkinje cell axons were myelinated in an organotypic cerebellar slice culture model, suggesting its important role in myelination (Schnadelbach et al., 2001). The cytoplasmic domain of cadherin can sequester β-catenin, thus modulating the levels of available β-catenin for the canonical Wnt signaling pathway, as well as β-catenin’s association with APC (adenomatous polyposis coli), a molecule implicated in the stabilization of microtubules (Hansen et al., 2008). Studies suggest that the Wnt signaling pathway could be an essential pro-myelination cascade (Tawk et al., 2011). Association of the cadherin cytoplasmic domain with α/β catenin and p120 is positively regulated by GSK3β and CaMKII, while it is negatively regulated by Src, Fer, abl and EGFR kinases (Nelson, 2008). Following cervical spinal cord unilateral hemisection, activation of the Wnt signaling pathway in corticospinal axons antagonizes regeneration via the Wnt1/Wnt5a/Ryk signaling complex (Liu et al., 2008; Lewallen et al., 2011; Tawk et al., 2011). The cytoplasmic cadherin domain also regulates both RhoGTPase activity, by interacting with p120, and actin dynamics, by interacting with α catenin (Cavallaro and Dejana, 2011). Conversely, Rho GTPase regulates the clustering of cadherins on the cell surface (Fukata and Kaibuchi, 2001; Grosheva et al., 2001). Cadherins can also bind to several growth factor receptors, including FGFR, and modulate their intracellular signaling, either by activating them in a ligand-independent mechanism, or by recruiting components for signaling units, which include adaptor proteins (Shc), kinases (Src, CSK) and phosphatases (SHP2, RPTPβ/η; Cavallaro and Dejana, 2011).

Nectins are the second group of calcium-independent CAMs that interact with themselves on the same cell (homophilical) or with nectin present on another cell (heterophilic; Takai et al., 2003). Further, a third group, nectin-like molecules (Necl), specific to nervous tissue, resemble nectins in structure and function and are expressed on axons (Kakunaga et al., 2005). Necl-1 has a strict neuronal expression in the cortex, retina, cerebellum and spinal cord (Kakunaga et al., 2005; Park et al., 2008). Necl-1 regulates time dependent critical aspects of myelination (Park et al., 2008; Zhu et al., 2013). Necl-1 knockout mice showed delayed axonal myelination of both the optic tract and spinal cord, which normalizes by 60 days post-birth (Park et al., 2008). Given a complex network of interactions and ligands, discerning the exact roles of CAMs in myelination would be an elaborate engagement, and has been excellently reviewed by Pollerberg et al. (2013).

The IgSF (immunoglobulin superfamily) is the fourth group of CAMs that do not depend on calcium and are present on axons. Growing axons express at least nine types of IgSF CAMs (NCAM1/2, L1-CAM, Contactin1/2, NRCAM, ALCAM, CHL1, and Neurofascin; Pollerberg et al., 2013). The IgSF members have a complex interaction network; they not only interact with themselves, but also with other CAMs in a homotypical or heterotypical manner. Extracellularly, IgSF CAMs have been noted to interact with CSPGs (neurocan, phosphacan), ECM components (Tenascin C and R, MMP14), β1 integrins, growth receptors (GDNF/GFRα1, FGF-R, TrkB), semaphorin receptors (Neuropilin 1, Plexin A1), ephrin receptors (Eph A 3/4/7), sodium and potassium channels (SCN1B, Kir 3.3), and with some unusual interacting partners, such as prion protein (PrPC), APP and extracellular GAPDH (Pollerberg et al., 2013). Intracellularly, IgSF CAMs routinely interact with cytoskeletal components such as ankyrin and the microtubule-associated protein, doublecortin (DCX; Rader et al., 1996; Kizhatil et al., 2002).

NCAM, one of the prominent members of IgSF CAM, is known to be involved in neural differentiation, axonal guidance and branching (Walsh and Doherty, 1997). Expression of unmodified NCAM persists during myelination (Bartsch et al., 1989). In contrast, post-translational modification of NCAM by the addition of sialic acid (PSA-NCAM) has been reported to negatively regulate myelination, with axons undergoing myelination only when they do not express PSA-NCAM, suggesting that inhibitory signals strongly regulate the myelination program (Charles et al., 2000; Fewou et al., 2007; Jakovcevski et al., 2007). The role of NCAM in myelination and neuroprotection could be more complex given its unconventional partners. For example, NCAM is known to interact with FGF and GDNF (GFRα1) receptors. Thus a trans-interaction with similar glial receptors can activate growth and differentiation pathways, driving differential myelination via FGF2 signaling (Jacobsen et al., 2008). Following transection SCI in rodents, NCAM levels increase in the dorsal spinal cord, motor neurons and corticospinal tract fibers (Tzeng et al., 2001). Investigations of contusive SCI in NCAM knockout mice revealed extensive neuronal apoptosis, decreased 5HT axon regeneration, and defective ERK and GAP43 signaling when compared to wild-type controls (Zhang S. et al., 2010). However, how NCAM, both native and surface modified, can alter ERK/GAP43 signaling and be neuroprotective in the injury scenario is unclear.

L1-CAM, a member of the IgSF CAM family, is intricately involved in axon growth and guidance during development (Cohen et al., 1998; Kenwrick and Doherty, 1998). Strong expression of L1-CAM has been found on unmyelinated optic nerve axons that are not in contact with glial cells, whereas it was absent from axon-glial contact regions (Bartsch et al., 1989). Trans-interactions of axonal L1-CAM can activate integrin signaling on glia, regulating axon-glia interactions and myelination (Silletti et al., 2000; Guseva et al., 2011). Transplantation of genetically engineered SCs producing L1, or secreting its chimeric form, L1-Fc, into the SCI lesion in mice, led to enhanced serotonergic fiber sprouting into, and across, the lesion (Lavdas et al., 2010). In addition, in transgenic mice that overexpressed L1 in neurons, severe contusion SCI studies suggested that L1-CAM could enhance catecholaminergic fiber regeneration and sprouting (Jakovcevski et al., 2013). It has been found that MBP, a myelin-associated protease present in both CNS and PNS myelin, is capable of cleaving L1-CAM to promote neuritogenesis (Lutz et al., 2015). This suggests that, similar to β1 integrins, myelin-associated proteins also can modulate CAM-mediated interactions.

Neurons express contactin, another member of the IgSF CAM family, which interacts heterophilically with L1-CAM, NRCAM and neurofascin (Falk et al., 2002). Since contactins do not have an intracellular domain, their signaling requires additional recruited molecules (Rios et al., 2000; Charles et al., 2002; Traka et al., 2002, 2003; Gautam et al., 2014). Contactin functions as a non-canonical notch ligand, and likely has numerous cell surface interaction partners (Hu Q. D. et al., 2006). Contactin2, another member of contactin superfamily, present on both SCs and OLs at the juxtaparanodal region, was recently discovered to be a BACE1 substrate, and an interaction partner of neuronal Caspr2 (a contactin-neurofascin interaction modulator) at paranodal areas (Rios et al., 2000).

Axons and glia harbor a host of molecules (including gliomedin, NrCAM and various isoforms of neurofascin), which take part in the maturation of the nodes of Ranvier on myelinated axons (Thaxton and Bhat, 2009). Myocilin, a Wnt and ErbB2/3 signaling regulator, interacts with gliomedin, NrCAM, and neurofascin (NF186; Kwon et al., 2013). Knocking out myocilin upregulates DLG1-PTEN in sciatic nerves (Cotter et al., 2010; Kwon et al., 2013). The DLG1-PTEN interaction negatively regulates peripheral myelin thickness, likely by stabilizing PTEN and decreasing Akt activation (Cotter et al., 2010). DLG1-mediated negative regulation of myelination was found to be transient, whereas a fine-tuning of PI3K-Akt-mediated mTOR signaling by another protein, DDIT4 (DNA damage-inducible transcript 4 protein), was found to lead to a sustained negative regulation of peripheral myelin thickness (Noseda et al., 2013). Conversely, the interactions of glial NrCAM and axonal contactin, as well as of glial neurofascin and axonal NrCAM have been shown promote neurite outgrowth in cultured tectal neurons (Morales et al., 1993; Grumet, 1997).

In summary, CAMs have dual roles in myelination and axonal growth that, at present, remain underinvestigated, particularly following injury. CAMs communicate with multiple downstream signaling pathways in a cell-specific manner. Conversely, axo-glial signaling pathways and myelin-associated proteins that share the same spatial domains can modulate CAM actions. Together, the advantages and disadvantages of CAMs, prompt a nuanced approach in their use for combinatorial therapies targeting myelination and axon regeneration.

### Chondroitin Sulfate Proteoglycans (CSPGs)

CSPGs constitute one of the important extrinsic factors limiting CNS axon regeneration following injury (Davies et al., 1997; Silver and Miller, 2004). OPCs, astrocytes and macrophages account for the predominant expression of CSPGs, which following SCI undergo tremendous modulation (Jones et al., 2002). CSPGs on the cell surface are often released from the membrane and become a part of the extracellular matrix (Carulli et al., 2005). The majority of CSPGs present in the CNS belong to the lectican family. Aggrecan, versican, neurocan, brevican, phosphacan, neuroglycan-D, NG2 and phosphacan constitute predominant CSPGs of the CNS (Carulli et al., 2005). Aggrecan is expressed primarily by neurons, versican by astrocytes, NG2 by OPCs and macrophages whereas neurocan and brevican are expressed by all neural cells (neurons, astrocytes and OPCs; Jones et al., 2002; Dyck and Karimi-Abdolrezaee, 2015). Structurally, CSPGs contain a protein core and glycosaminoglycan (GAG) side chains that routinely undergo excessive branching or modifications like sulfation (Carulli et al., 2005). Modifying the GAG chain lengths (using enzyme Cha’se ABC) or their sulfation modulates CSPG permissiveness for axon regeneration (Zuo et al., 1998; Wang et al., 2008).

CSPGs interact with a multitude of axonal and growth cone surface receptors such as Nogo receptor 1 (NgR1), Nogo receptor 2 (NgR2), leukocyte common antigen (LAR) and protein tyrosine phosphase σ (PTPσ). Possibly with more players (such as p75NTR), CSPG-receptor interaction can negatively regulate axon regeneration by activating the Rho/ROCK signaling pathway (Dergham et al., 2002; Monnier et al., 2003). A recent study suggests that extracellular CSPGs, by an as yet unknown mechanism, can regulate local intra-axonal translation of RhoA, thus ensuring an enhancement of Rho/ROCK signaling in the axon (Walker et al., 2012). Interestingly, studies implicate NG2 (expressed on OPCs) to be the predominant CSPG following dorsal hemisection SCI (Plant et al., 2001; Jones et al., 2002). NG2-positive OPCs surround corticospinal axons within the injury environment after SCI (Jones et al., 2002). Based upon this interaction, NG2 could then serve as an important reason for impaired axon regeneration following injury. However, even though NG2 *per se* inhibits axon growth, both postnatal and adult NG2 expressing cells are growth permissive (Dou and Levine, 1994; Ughrin et al., 2003; Yang et al., 2006; Busch et al., 2010). This paradox could be due to the co-expression of axonal growth permissive substrates (e.g., laminin and fibronectin) on NG2 positive cells (Yang et al., 2006). In addition, SCs have been shown to express CSPGs that are sensitive to treatment with chondroitinase (Cha’se) ABC, and which retard DRG axon growth (Kuffler et al., 2009). Together these findings suggest that glial CSPGs, both membrane-bound and ECM-associated, can interact with several membrane receptors on the axon, growth cone and soma to modulate the axon growth machinery. Whether membrane bound CSPGs can activate distinct molecular pathways in axons as compared to those associated with the ECM remains unknown.

OPCs show an increase in PTP expression during their differentiation (Ranjan and Hudson, 1996). CSPGs negatively regulate OL process outgrowth and the OL myelination program by interacting with PTPσ (Pendleton et al., 2013). In one study, aggrecan, neurocan and NG2, all inhibited OL process outgrowth, and downregulated MBP expression, and this effect was reversed by Cha’se ABC (Pendleton et al., 2013). In addition, a combination of CSPGs (neurocan, versican, phosphacan and aggrecan) led to a stronger phenotype of OL process inhibition and MBP expression in OLs (Siebert and Osterhout, 2011). Treatment of OPCs with Cha’se ABC also enhanced their spontaneous differentiation towards O4-positive OLs (Karus et al., 2016). This effect of Cha’se ABC could be mediated by its ability to eliminate phosphacan/RPTPβ/ζ from the OPC surface (Karus et al., 2016). Moreover, Cha’se ABC stimulated neural precursor cells in the mouse spinal cord to differentiate into OLs in large numbers (Karus et al., 2016). Intracellularly, CSPG-mediated changes in the outgrowth and alterations in the myelination program of OPCs and OLs was mediated by PTPσ driven Rho/ROCK activation (Pendleton et al., 2013). Together these findings suggest that similar molecular pathways activate CSPG-mediated axon growth inhibition and the OL myelination program. Therefore, it is important that signaling intermediaries/downstream of Rho/ROCK are identified so as to permit future therapeutic targeting of CSPGs’ differential effects on myelination and axon regeneration.

## The Physical Properties of Axons and the Congruence of Myelination and Axonal Growth

### Axonal Caliber and Radial Growth of Axons

Axon diameters vary in the CNS (0.1–24 μm), and accordingly, their cross sectional area and volume (Barazany et al., 2009; Perge et al., 2012). In an elegant experiment, Voyvodic showed that altering the target size of postganglionic, unmyelinated sympathetic axons altered their caliber, as well as the responses of SCs towards them, suggesting a strong correlation between axonal caliber and myelination (Voyvodic, 1989). As a corollary to Voyvodic’s (1989) work, axons that differ in their caliber above the physiological limit (100 nm) appear to reflect the information they carry to and from their target (Faisal et al., 2005; Perge et al., 2012). In the PNS, axons less than 700 nm in diameter are not myelinated but are ensheathed by a SC, or reside in a Remak bundle (Garbay et al., 2000). Such a threshold for ensheathment and myelination is much lower in the CNS, where OLs myelinate axons of around 200 nm diameter and axon-like nano fibers of ≥ 400 nm in diameter *in vitro* (Waxman and Bennett, 1972; Lee et al., 2013). OLs have been observed to produce myelin components without the presence of axons, but intriguingly, in *in vitro* co-cultures with neurons, OLs myelinate only axons even when the dendrite diameters vary from 200 to 8000 nm, suggesting a role for axon-specific signals in myelination (Ulfhake and Cullheim, 1981; Dubois-Dalcq et al., 1986; Claiborne et al., 1990; Lubetzki et al., 1993). Indeed, SCs that do not myelinate cervical sympathetic axons were observed to myelinate sural nerve axons, which routinely undergo myelination, reiterating axon-specific signals for myelination (Aguayo et al., 1976). Intriguingly, a linear relation between the cross-sectional area of the myelin sheath and axon diameter (see G-ratio) was observed, suggesting a role for the axonal surface in regulating myelin sheath volume (Berthold et al., 1983; Paus and Toro, 2009). G-ratio is the ratio of axonal diameter to fiber diameter (including myelin sheath), used as a measure for optimal myelination. An axon can influence its myelination by communicating its information load via specific molecular signals to their ensheathing cells (Fraher and Dockery, 1998). Supporting this view, in the PNS, axons directly control the thickness of their myelin sheath via the regulation of the expression of NRG1 type III on the axonal membrane (Michailov et al., 2004; Taveggia et al., 2008).

Conversely, myelination can affect axonal caliber in the PNS and the CNS, effectively modulating their radial growth. Myelinated axons have been consistently observed to have a larger diameter than unmyelinated axons (Duncan, 1934; Matthews and Duncan, 1971; Hoffman et al., 1984; de Waegh et al., 1992). Myelination increases the cross sectional area of the myelinated axonal segment up to 45% more than the unmyelinated segment of the same axon (Monsma et al., 2014). Structurally, medium and heavy neurofilaments (NF-M and NF-H) principally determine the caliber of the axon, and a defective NF phenotype presents with abnormal axonal caliber (Hoffman et al., 1987; Cleveland et al., 1991; Muma and Cork, 1993). Therefore, NF-M and NF-H could be among the putative molecules responding to glial-derived, radial axon growth signals. In SC-DRG co-culture experiments, myelinated axonal segments were observed to contain nearly 42% more NF-M as compared to unmyelinated segments of the same axon (Monsma et al., 2014). SC-DRG co-culture experiments have suggested that the factor responsible for an enlarged diameter of myelinated axons was not secreted but rather a cell-to-cell contact molecule (Windebank et al., 1985). So far, myelin-associated molecules such as MAG, PLP, PMP22, MBP, and sulfated and non-sulfated galactolipids (sulfatide/GalC) have been suggested to play a role in axon-glial communication and adhesion, to modulate the radial growth of axons (de Waegh and Brady, 1990; Smith et al., 2013). MAG is a component of non-compact PNS myelin in the periaxonal membrane (Trapp and Quarles, 1984; Patzig et al., 2011). Mice having a complete absence of MAG, and rats having a mutation in MBP [LES (long Evans shaker)], show: (1) decreased NF density in sciatic nerve axons; (2) decreased NF phosphorylation; (3) decreased axonal caliber and, (4) progressive axonal loss by Wallerian degeneration (Yin et al., 1998; Smith et al., 2013). Addition of soluble MAG-Fc to DRG neuronal cultures was found to increase the phosphorylation of NF-H, and negatively regulate axonal degeneration induced by vincristine, thus supporting the role of MAG in modulating axonal caliber (Nguyen et al., 2009). However, so far very few studies have addressed the mechanistic pathway connecting MAG or other myelin-associated proteins to the radial growth of axons. Growth cone collapse and the mechanisms thereof have received a higher emphasis to date. MAG and OMgp also cause longitudinal growth inhibition of axons by synergizing with another MAI, Nogo-A, which binds to NgR1 to bring about growth cone collapse (Cafferty et al., 2010). Lipid-sulfatides are a novel class of MAIs that presumably work through the canonical MAI pathway leading to growth cone collapse in a Rho/ROCK dependent manner (Winzeler et al., 2011).

Unraveling the mechanisms involved in the post-translational modulation of NFs from glial derived signals remains an ongoing pursuit. Initial thinking was that in the myelinated segments of axons, heavily phosphorylated NF tail domain KSP motifs exert repulsive forces on neighboring NFs by long side-arms and hence spread apart. Whereas, in unmyelinated segments they are less phosphorylated and therefore tightly packed (Hisanaga and Hirokawa, 1988; Mukhopadhyay et al., 2004; Sihag et al., 2007). Previous work has unequivocally shown that the phosphorylation of NFs not only promotes NF-NF interactions, but also affects their ability to associate with molecular motors such as kinesin, dynein and myosinV (Yabe et al., 2000; Xia et al., 2003; Motil et al., 2006; Kushkuley et al., 2009). On the contrary, experiments conducted with a substitution of serine in the KSP motifs of NF-M with phosphorylation-incompetent amino acids demonstrated radial axonal growth to be independent of the NF-M-KSP phosphorylation motif, but dependent on the C terminal region of NF-M, thus prompting a relook at the prevailing model of radial axonal growth (Garcia et al., 2009). Recent evidence suggests that the C-terminal domains of NF-M and NF-H can play a role in the stabilization of NFs by modulating its proteolysis (Rao et al., 2012). Unmyelinated peripheral axons do express components of all three different forms of the proteolytic machinery (ubiquitin-proteasome system, lysosomes and autophagy), which can be regulated by neurotrophins (e.g., NGF; Frampton et al., 2012). Taken together, post-translational modification of NFs seems to play a significant role in modulating axonal caliber. However, mechanistic details of how MAG, or any other myelin-associated molecule, can modulate axonal caliber remains largely speculative. GSK3α/β, CDK5, MAPK, SAPK/JNK, CKI/II are some of the implicated phosphokinases that can phosphorylate NFs, whereas PP1, PP2A and PTP1B are some of the phosphatases known to reverse NF phosphorylation (Shea et al., 2004; Snider and Omary, 2014). The proposed hypothesis is that MAG phosphorylates NFs by interacting with the low affinity neurotrophin receptor (p75NTR) to activate Raf/MEKK_1/2_/ERK_1/2_ via NRAGE. In addition, p75NTR/NRAGE can inhibit PKA, leading to increased Rho and inactivation of p35/Cdk5. This could produce the decreased Cdk5 phosphorylation of NF as well as decreased Cdk5-mediated inhibition of ERK_1/2_. The signaling from MAG/p75NTR/NRAGE could then fine-tune the phosphorylation status of NF-M and NF-H (Garcia et al., 2003). However, unequivocal confirmation that MAG can recruit these signaling pathways remains to be shown. Recently, in a work identifying new players connected to NF phosphorylation, a study found that the loss of β1 integrin could lead to the phosphorylation of Tau at Ser262 (Lei et al., 2012). In a separate study, MARK4/3 (microtubule-associated regulating kinase) expression correlated with pTau (Ser262) levels in granulovacuolar degeneration bodies of the Alzheimer’s diseased brain, which shows progressive defects in NF phosphorylation (Lund et al., 2014). Whether myelin-associated proteins could mediate trans-regulation of the axonal proteolytic system remains to be explored.

In summary, post-translational modifications of NF may have differential effects on its stability and its association with other NF molecules as well as transport motors, depending on the specific kinase/phosphatase/proteolytic machinery within the local environment. Specific glial and myelin-associated molecules that regulate axonal caliber and their partners, along with the related signaling network upon the axonal membrane, are a significant gap in our understanding of axon-glial signaling that needs further attention.

### Myelination and Axonal Transport

The trembler mouse, which displays hypomyelination in the PNS, also shows an altered axonal transport (de Waegh and Brady, 1990). The trembler mouse has a point mutation in PMP22 that encodes a peripheral-myelin enriched protein (Suter et al., 1992). Decreased myelination of the sciatic nerve in the trembler mouse was associated with increased NF transport (1.73 mm/d in trembler vs. 1.56 mm/d in control; de Waegh and Brady, 1990). In contrast, a slower NF transport velocity of 0.16 mm/d occurred in DRG neuron axonal segments that were SC-myelinated, compared to 0.22 mm/day in the unmyelinated segments (Monsma et al., 2014). It is interesting to note that the NF transport rate has been reported to be quite heterogeneous, depending on the cell type and the analysis method employed (Shea and Chan, 2008). There is a considerable debate on the role of phosphorylation of the NF tail domain in restricting NF transport, since the evidence so far supports the role of NFs in the radial growth of axons (Yuan et al., 2006; Sihag et al., 2007; Shea and Chan, 2008).

Interestingly, addition of soluble MAG to the axonal compartment of rat cortical cultures promoted the accumulation of detyrosinated tubulin, and decreased tyrosinated tubulin in a NgR-independent, but GalNAc-T dependent manner (Nguyen et al., 2009). Though exciting, this finding needs to be reconfirmed since the antibody used by Nguyen et al. (2009) to detect de-tyrosinated tubulin (Chemicon: AB3210) was raised against β-tubulin and the antibody used to detect tyrosinated tubulin (Sigma: T9028) was raised against α-tubulin. Post-translational modification of tubulin-dimers (typically α-tubulin) by the addition or deletion of tyrosine is known to regulate its association with kinesins and many plus-end tracking proteins (+TIPS), including p150Glued, a member of dynein-dynactin retrograde motor complex, and CLIP-170, a CAP-Gly domain-containing protein implicated in injury signaling (Lomakin et al., 2009; Lloyd et al., 2012; Gumy et al., 2013; Prota et al., 2013; Schneider et al., 2015). In addition, other players can also be involved in modulating the axonal cytoskeleton. Work has pointed towards notch signaling involvement in the stabilization of microtubules through transcriptional regulation of the microtubule severing protein, spastin (Ferrari-Toninelli et al., 2008). Thus, post-translational modulation of axonal tubulin by OL/SC surface molecules opens a completely new chapter in glia-modulated structural and functional aspects of the axonal cytoskeleton.

The precise signaling mechanisms involved in the regulation of tubulin tyrosination remain unknown. Post-translationally (phosphorylation) controlled tubulin tyrosine ligase (TTL) activity regulates tubulin tyrosination (Idriss, 2000). Loss of TTL leads to severe defects in microtubule-dependent retrograde transport, specifically after axonal injury (Idriss, 2000; Song et al., 2015). Mechanistic details of TTL phosphorylation remain elusive, but MKP-1 can be one such putative candidate. A novel MKP-1 (Map Kinase Phosphatase-1; MKP-1^ASA^) was constructed by Jeanneteau et al. (2010) by mutating its RRR motif in the MAPK binding domain, which eliminated the binding of MKP-1 to ERK_1/2_ and p38 MAPK, but selectively retained the MKP-1/JNK-1 interaction (Jeanneteau et al., 2010). Ectopic expression of MKP-1^ASA^ in the postnatal rat cortex decreased JNK-1 activity and increased tyrosinated-α-tubulin in both excitatory neurons and interneurons (Jeanneteau et al., 2010). Moreover, different isoforms of JNK along with their substrates, stathmins (e.g., SCG10, SCLIP), are known to be crucial for microtubule stability and axon growth (Tararuk et al., 2006; Barnat et al., 2010). Whether myelin-associated proteins selectively trans-activate the MKP-1/JNK pathway to regulate axonal TTL is an open question. A study suggested that retrogradely transported TrkB-pERK could regulate the transient expression of inducible MKP-1 (Jeanneteau et al., 2010). It is therefore tempting to speculate that regeneration-associated signaling and myelination could mutually influence each other through cytoskeletal components. Elucidating these mechanisms could provide novel avenues for regeneration research.

### Adaptive Myelination

Neuronal activity influences oligodendrogenesis, OL differentiation, myelination, and myelin thickness (Gyllensten and Malmfors, 1963; Tauber et al., 1980; Barres and Raff, 1993; Stevens et al., 1998; Gibson et al., 2014). Decreasing the neuronal activity just prior to developmental myelination of the optic nerve, with the application of a sodium channel blocker (tetrodotoxin), has been shown to produce a 60% decrease in the optic nerve myelination without affecting OPC numbers (Demerens et al., 1996). In contrast, exposure of developing zebrafish to the GABA antagonist pentylenetetrazole (PTZ), in the bathing medium, not only increased neuronal activity, but also led to a 40% increase in the number of myelin sheaths made by OLs upon reticulospinal axons of the ventral spinal cord, without any major changes in OL numbers (Mensch et al., 2015).

Emerging evidence suggests that OLs can sense neuronal activity and make decisions regarding the ensheathment of axons (Hines et al., 2015). In a study involving spinal cord samples of zebrafish embryos, individual OLs were seen to lay the myelin sheath within 5 h of contact with the target axons that were electrically active (Czopka et al., 2013). Blocking sodium channels in zebrafish using TTX, biased the stabilization, extension and maintenance of myelin sheaths away from highly active axons, without affecting OL differentiation. Further experiments highlighted that vesicle-associated membrane protein (VAMP2) dependent extra-synaptic axonal secretion regulated the maintenance of OL ensheathment (Wake et al., 2011; Hines et al., 2015). Inhibition of synaptic vesicle release in developing zebrafish could reduce the average number of myelin sheaths for OLs by 30%, OL numbers by 10% and the number of myelinated reticulospinal axons by 40% (Mensch et al., 2015). Optogenetic stimulation of the premotor cortex (M2) in a transgenic mice (heterozygous for Thy1::ChR2) led to similar findings of activity-dependent myelination with functional modulation (Gibson et al., 2014).

Extrasynaptic neurotransmitter release from neurons is known to modulate purinergic and glutamatergic signaling in glia (Stevens et al., 2002; Kukley et al., 2007; Ziskin et al., 2007; Thyssen et al., 2010; Wake et al., 2011). Extrasynaptic glutamate release from DRG neuronal cultures is vesicular, whereas ATP release appears to be non-vesicular (Wake et al., 2011). Sensory neuronal activity activates the PI3K/Akt/CREB pathway in SCs by an ATP-dependent mechanism, possibly involving P2Y purinergic receptors, which results in an inhibition of the proliferation and differentiation of SCs (Stevens and Fields, 2000). On the contrary, priming by adenosine, not ATP, promotes OPC differentiation and subsequent myelination of DRG in co-culture studies (Stevens et al., 2002). The pro-myelinating features of purinergic signaling have been found to operate predominantly through the OPC somata, which is not in direct contact with the axon underneath (Wake et al., 2011). Previous studies with neuron-OPC co-cultures hinted towards the existence of soluble signals from neurons that could affect the way OPCs regulate myelin biogenesis (Simons and Trajkovic, 2006). This work found that neuronal signals reversed the cholesterol-dependent endocytosis within OPCs and triggered the fusion of PLP/DM20 (compact intermodal myelin proteins) carrying late endosomes/lysosomes (Simons and Trajkovic, 2006). DRG:OPC co-culture studies revealed that glutamate release from axons forms the main hub for axon-glial signaling through its interaction with metabrotopic (mGluR) and ionotropic (NMDR) glutamate receptors on the OPC processes (Wake et al., 2011). More recent studies have highlighted the role that AMPA/Kainate glutamate receptors play in regulating OPC response to vesicular glutamate release from axons/*de novo* synapses (Fannon et al., 2015; Gautier et al., 2015). In DRG:OPC co-cultures, electrical stimulation of neurons produced an increased clustering of phosphorylated Fyn kinase at cholesterol-rich micro domains on OPCs, as visualized by the transferrin receptor (TfR; Wake et al., 2011). Phosphorylated Fyn kinase has been reported to, in turn, phosphorylate heterogeneous nuclear ribonucleoprotein A2 (hnRNPA2), which is located within the OPC processes. Fyn phosphorylation of hnRNPA2 releases the repression on MBP mRNA, making it available for local translation (White et al., 2008). Indeed, neuronal activity enhances the local translation of MBP in the OPC processes (Wake et al., 2011).

There is emerging evidence that the activity-dependent regulation of OPC programming and their subsequent ability to myelinate axons might have many more players than previously thought. Intriguingly, in DRG-OPC co-cultures, NRG (NRG1β, soluble) and BDNF were able to switch the OPC myelination program from activity-independent to activity-dependent (Lundgaard et al., 2013). N-methyl-D-aspartate receptors (NMDA) on OPCs mediate NRG-induced OPC maturation and myelination (Lundgaard et al., 2013). The activity-dependent myelination program in OPCs, in the presence of NRG, was inhibited by an antibody that blocked β1 integrin (Lundgaard et al., 2013). Additionally, sodium-dependent glutamate transporters (excitatory amino acid transporters, EAAT1/2/3) can mediate glutamate signaling via CaMKIIβ, to modulate OPC morphology and myelin biogenesis (Martinez-Lozada et al., 2014). This highlights that adaptive myelination could be further subjected to *cis*-regulation on myelinating glia.

Studies have also suggested that activity-dependent axon-glial/neuron-glial communication could be mutual. Neuronal activity can promote Rab35-mediated exosome release from OPCs, in an AMPA/NMDA-dependent manner. Neurons internalize these exosomes by taking them up via axonal and somatodendritic compartments (Fruhbeis et al., 2013). The exosomes contain myelin component proteins (e.g., PLP) and heat shock proteins, which provide ER and starvation protection to neurons. Neuronal activity has also been identified to directly regulate axonal growth by modulating GAP-43 transcription and post-translational regulation, in addition to altering key signaling pathways (e.g., Notch) that might have a profound impact on regeneration and neuropathic pain post-injury (Cantallops and Routtenberg, 1999; Howe, 2003; Alberi et al., 2011). Neuronal activity regulates neuronal notch signaling in an Arc/Arg3.1-dependent manner (Sestan et al., 1999; Alberi et al., 2011). In adult NG2^+^ OPCs, neuronal activity was also reported to promote ADAM10 dependent cleavage of NG2, one of the extrinsic inhibitory factors for axonal regeneration post-injury (Dou and Levine, 1994; Ughrin et al., 2003; Sakry et al., 2014).

Taken together, these findings suggest that axon-glia communication is dynamic, offering a potential untapped target for remyelination therapeutics. The molecular machinery in adaptive myelination includes those responding to both paracrine (NRG1 and neurotransmitter) as well as local signaling (β1 integrins). Further experiments to understand the precise molecular nodes required for the provision of an on-demand stimulation of the myelination program remain to be undertaken.

## Summary

Nervous system injury, such as trauma to the spinal cord, poses a challenge to the intricately laid myelin structure surrounding axons. The loss of the myelinating glia or myelin damage leads to eventual myelin degradation and denuding of the axon, then axonal retraction of cut axons or conduction block within those spared, but demyelinated axons (Olby and Blakemore, 1996; Grossman et al., 2001; Guest et al., 2005; Hagg and Oudega, 2006; Lytle and Wrathall, 2007; Ek et al., 2012; Seidl, 2014). Axon-glial communications during the myelination process affect two critical decisions in axonal growth. The first of these are the signals for ensheathment and myelination of the axon that originate in axons themselves, while the second are signals that inhibit longitudinal growth to promote radial growth of the axon that originate from glia. Studies have highlighted the importance of PI3K/Akt in the myelination program and ERK/p38MAPK in axonal growth. However, accumulating evidence also suggests that outcomes of Akt/MAPK signaling cascades can be tremendously influenced by various *cis* and *trans* regulators on axon-glial surfaces, myelin components and co-activated signaling pathways. In addition, neuronal activity regulates the nature of axon-glial communication. Multiple molecules, canonically known to be important for myelination and axonal growth (e.g., notch, NRG, integrins and L1-CAM etc.) respond actively to neuronal activity. On certain occasions, surface ligands themselves can alter the downstream canonical signaling. In summary, various axon-glial communications regulate the myelination program and axonal cytoskeleton, by modulating the critical nodes of intracellular signaling pathways and the neuron-glia transcriptional network. This highlights a need for combinatory approaches that modulate both surface axon-glial communication and intracellular signaling to effectively control the process of axon growth and myelination.

There is an enormous need to revisit axon-glial signaling in clinically relevant nervous system injury models to generate an index of perturbed pathway components and axon-glial surface molecules. Specifically, a comprehensive understanding of the signaling pathways that are mediated through myelin components, both intact and debris, in the regulation of radial axon growth is needed. Further investigation into how these signals intersect with those pathways implicated in the initiation and elongation of axons during longitudinal growth, may offer unique opportunities for targeting the temporal regulation of endogenous and exogenous (implanted) glia interactions, as well as their communication with the injured axon to maximize axon growth to appropriate targets. Given the amount of glial cells the regenerating axons encounter in an injury scenario, it would therefore be appropriate to negatively target glial-derived axon growth inhibitory signals, and positively amplify longitudinal axon growth promoting signals for effective regeneration. Understanding this critical network holds the key to prime selectively the myelination program or to promote axonal growth.

## Author Contributions

DDP designed the outline and edited the manuscript, SNRR wrote the manuscript and edited the manuscript.

## Funding

We would like to acknowledge research support from John M. and Jocelyn H.K. Watkins Distinguished Chair in Cell Therapies (DDP).

## Conflict of Interest Statement

The authors declare that the research was conducted in the absence of any commercial or financial relationships that could be construed as a potential conflict of interest. The reviewer SK-A and handling Editor declared their shared affiliation, and the handling Editor states that the process nevertheless met the standards of a fair and objective review.
